# Race and Ethnic Group Differences in Social Engagement Among Older Adults

**DOI:** 10.1093/geroni/igab046.1056

**Published:** 2021-12-17

**Authors:** Lien Quach, Uyen-Sa Nguyen, Van Pham, Jeffrey Burr

**Affiliations:** 1 University of Massachusetts Boston, Newton, Massachusetts, United States; 2 University of North Texas Health Science Center (UNTHSC), School of Public Health (SPH), Fort Worth, Texas, United States; 3 Center for Promotion of Advancement of Society, Hanoi, Ha Noi, Vietnam; 4 McCormack Graduate School, Boston, Massachusetts, United States

## Abstract

Social engagement is considered crucial for older adults’ well-being, generating social capital, connecting them to information about healthy lifestyles, and providing coping strategies for addressing daily challenges. Little is known about race and ethnic disparities regarding social engagement. This study examines the relationship between race, Hispanic ethnicity, and social engagement among community-dwelling adults age 65 or older. Data are taken from the Health and Retirement Study (2014) (n=6,221). Race and ethnic status are measured as: non-Hispanic white, non-Hispanic black, non-Hispanic “Asians and other race,” and Hispanic (any race). Social engagement includes frequency of contact with friends and family and participation in social activities (e.g. volunteering and attending religious services). Covariates included age, sex, education, number of co-morbidities, and alcohol consumption. Linear regression analyses were performed using SAS 9.4. The mean age was 74.6, and sixty percent of the sample was female. Race and ethnic distribution were 78.6% non-Hispanic white, 11.9% non-Hispanic black, 7.89% Hispanics, and 1.7% non-Hispanic “Asians and other race.” The mean score for our social engagement index was 3.3 (range 0-6). Hispanic persons, Asian persons, and persons from other race groups had lower social engagement compared with non-Hispanic white persons [β:-0.29, p<.0001; β:-0.27, p=0.04 respectively), after adjusting for covariates. These race and ethnic group differences in social engagement likely contribute to well-document health disparities in later life. Understanding racial and ethnic disparities in social engagement and the factors that create these differences can help identify appropriate social intervention programs regarding improving the well-being of all older adults.

